# Terpenoids and Their Biosynthesis in Cyanobacteria

**DOI:** 10.3390/life5010269

**Published:** 2015-01-21

**Authors:** Bagmi Pattanaik, Pia Lindberg

**Affiliations:** Department of Chemistry—Ångström, Uppsala University, Box 523, SE-751 20 Uppsala, Sweden; E-Mail: bagmi.pattanaik@kemi.uu.se

**Keywords:** terpenoids, isoprenoids, cyanobacteria, MEP pathway, genetic engineering

## Abstract

Terpenoids, or isoprenoids, are a family of compounds with great structural diversity which are essential for all living organisms. In cyanobacteria, they are synthesized from the methylerythritol-phosphate (MEP) pathway, using glyceraldehyde 3-phosphate and pyruvate produced by photosynthesis as substrates. The products of the MEP pathway are the isomeric five-carbon compounds isopentenyl diphosphate and dimethylallyl diphosphate, which in turn form the basic building blocks for formation of all terpenoids. Many terpenoid compounds have useful properties and are of interest in the fields of pharmaceuticals and nutrition, and even potentially as future biofuels. The MEP pathway, its function and regulation, and the subsequent formation of terpenoids have not been fully elucidated in cyanobacteria, despite its relevance for biotechnological applications. In this review, we summarize the present knowledge about cyanobacterial terpenoid biosynthesis, both regarding the native metabolism and regarding metabolic engineering of cyanobacteria for heterologous production of non-native terpenoids.

## 1. Introduction

Terpenoids, or isoprenoids, are a large family of compounds including carotenoids, tocopherol, phytol, sterols and hormones. There are tens of thousands of known terpenoid compounds, and likely many more that have not yet been described. In all living organisms, terpenoids play a role in respiration chain electron transport (ubiquinone and menaquinone) as well as in cell wall and membrane biosynthesis and stability (bactoprenol, hopanoids in bacteria and sterols in plants). Plants are one of the major sources of terpenoid diversity. Terpenoids are vital for the growth and survival of photosynthetic organisms, since they play an essential role in conversion of light into chemical energy and for assembly and function of photosynthetic reaction centers (chlorophylls, bacteriochlorophylls, rhodopsins and carotenoids). Other known functions of plant terpenoids include important roles in stress response or in defense mechanisms [1,2].

With such wide range of biological functions, terpenoids have extensive applications in the fields of pharmaceuticals, cosmetics, colorants, disinfectants, fragrances, flavorings and agrichemicals. Several terpenoids have also been used as drugs to benefit human health, such as artemisinin used as an antimalarial drug [3]. Paclitaxel, known as taxol, is an effective anti-cancer agent [4]. Avicins [5] and parthenolide [6] have been shown to reduce growth of tumor cells. Betulinic acid was found to exhibit anti-HIV-1 activity [7]. Carotenoids, such as lycopene and astaxanthin, are the focus of research into their potential benefits for human health and treatment of disease [8,9].

Despite their diversity in structure and function, all terpenoids are made from the same five-carbon building blocks, isopentenyl diphosphate (IDP) and dimethylallyl diphoshate (DMADP). IDP and DMADP in turn originate from either of two distinct pathways. The mevalonate (MEV) pathway uses acetyl-CoA as a substrate and converts this in six steps, where mevalonate is one intermediate, to IDP, which can be interconverted to DMADP by an isomerase, see [10], and references therein). This pathway operates in eukaryotes, archaea and some bacteria, and was long thought to be the exclusive route to formation of terpenoids in all organisms. In 1993, this paradigm was overturned by the discovery of an alternate route for terpenoids biosynthesis in bacteria, which uses pyruvate and glyceraldehyde-3-phosphate as substrates to form both IDP and DMADP [11,12]. The new pathway is now commonly referred to as the MEP pathway, for the intermediate methylerythritol-4-phosphate. The MEP pathway was subsequently found to be the most common route to formation of terpenoids in eubacteria as well as being used in plant plastids and in algae [13,14] in parallel with a cytosolic MEV pathway.

In cyanobacteria, only a few studies have addressed the MEP pathway and the basis for terpenoids biosynthesis. There is, however, a large diversity of terpenoid compounds produced by cyanobacteria, notably pigments with different functions and characteristics but also other compounds with a variety of structures and functions. In this review, we will describe the knowledge about terpenoid biosynthesis in cyanobacteria in general, and also describe known pathways and functions for specific terpenoids compounds, both those that are naturally occurring and those that have been heterologously produced in cyanobacteria.

## 2. Specific Enzymes Involved in the MEP Pathway

**Figure 1:**
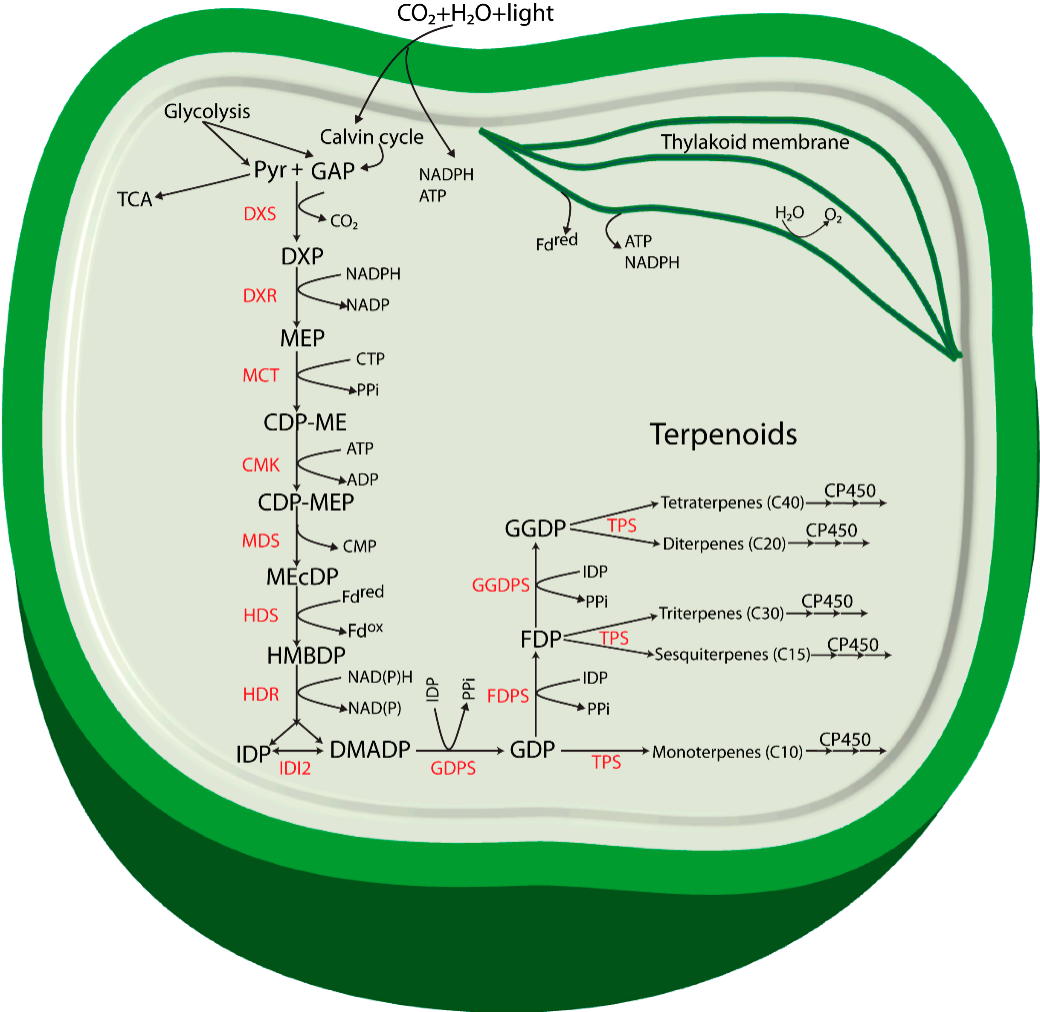
Proposed terpenoid biosynthesis via the methylerythritol-4-phosphate (MEP) pathway in cyanobacteria. Abbreviations: Pyr: Pyruvate; GAP: glyceraldehyde 3-phosphate; DXP: 1-deoxy-d-xylulose 5-phosphate; MEP: methylerythritol-4-phosphate; CDP-ME: 4-(cytidine 5'-diphospho)-2-*C*-methyl-d-erythritol; CDP-MEP: 2-phospho-4-(cytidine 5'-diphospho)-2-*C*-methyl-d-erythritol; MEcDP: 2-*C*-methyl-d-erythritol 2,4-cyclodiphosphate; HMBDP: 4-hydroxy-3-methylbut-2-enyldiphosphate synthase; IDP: isopentenyl diphosphate; DMADP: dimethylallyl diphosphate; GDP: geranyl diphosphate; FDP: farnesyl diphosphate; GGDP: geranylgeranyl diphosphate; DXS: 1-deoxy-d-xylulose 5-phosphate synthase; DXR: 1-deoxy-d-xylulose 5-phosphate reductoisomerase; MCT: 2-*C*-methyl-d-erythritol 4-phosphate cytidylyltransferase; CMK: 4-(cytidine 5'-diphospho)-2-*C*-methyl-d-erythritol kinase; MDS: 2-*C*-methyl-d-erythritol 2,4-cyclodiphosphate synthase; HDS: 4-hydroxy-3-methylbut-2-enyl diphosphate synthase; HDR: 4-hydroxy-3-methylbut-2-enyldiphosphate reductase; IDI: isopentenyl diphosphate isomerase; GDPS: geranyl diphosphate synthase; FDPS: farnesyl diphosphate synthase; GGDPS: geranylgeranyl diphosphate synthase; TPS: terpene synthase; CP450: cytochrome P450 monooxygenase.

The MEP pathway, as described for *Escherichia coli* [12], starts with condensation of glyceraldehyde 3-phosphate (GAP) and pyruvate to form 1-deoxy-d-xylulose 5-phosphate (DXP). Formation of DXP is an irreversible reaction catalyzed by the enzyme 1-deoxy-d-xylulose 5-phosphate synthase (DXS) with release of one molecule of CO_2_ (Figure 1). Involvement of DXS in the pathway was functionally analyzed for the first time in *E. coli* [15] and was also investigated in the cyanobacterium *Synechococcus leopoliensis* SAUG 1402-1 [16]. In plants, DXS plays a major role in the overall regulation of the pathway [17].

The enzyme 1-deoxy-d-xylulose 5-phosphate reductoisomerase (DXR) catalyzes a reaction where DXP is reduced to form the second intermediate MEP. The formation of MEP takes place by an intramolecular rearrangement of the carbon backbone and a NADPH-dependent reduction of the intermediate 2-*C*-methyl-erythrose 4-phosphate [18]. DXP is also found to be involved in thiamine and pyridoxol synthesis in bacteria and in plant chloroplasts [15,19,20]. The third intermediate of the pathway, 4-(cytidine 5'-diphospho)-2-*C*-methyl-d-erythritol (CDP-ME) is formed from MEP in a CTP-dependent reaction (cytidylation) catalyzed by 2-*C*-methyl-d-erythritol 4-phosphate cytidylyltransferase (MCT). The hydroxyl group in the C2 position of CDP-ME is further phosphorylated in an ATP-dependent manner by 4-(cytidine 5'-diphospho)-2-*C*-methyl-d-erythritol kinase (CMK) to form 2-phospho-4-(cytidine 5’-diphospho)-2-*C*-methyl-d-erythritol (CDP-MEP), the fourth intermediate. CDP-MEP is subsequently converted to 2-*C*-methyl-d-erythritol 2,4-cyclodiphosphate (MEcDP), the fifth intermediate of the pathway, in a reaction catalyzed by 2-*C*-methyl-d-erythritol 2,4-cyclodiphosphate synthase (MDS). It was recently discovered that MEcDP is a key metabolite and has an important role in regulation of the pathway [21], and references therein). MEcDP is reduced by 4-hydroxy-3-methylbut-2-enyl diphosphate synthase (HDS) to form 4-hydroxy-3-methylbut-2-enyldiphosphate (HMBDP). In plants, this step is dependent on reduced ferredoxin [22], and electron transfer from ferredoxin to HDS has also been demonstrated for enzymes from the cyanobacterium *Thermosynechococcus elongatus* BP-1 [23].

HMBDP is converted by HMBDP reductase (HDR) into a mixture of IDP and DMADP. The function of HDR and its importance in the terpenoid pathway was first described in a study in the cyanobacterium *Synechocystis* sp. PCC 6803 (hereafter referred to as *Synechocystis*) [24]. The researchers observed that *Synechocystis* cells with a mutation in the *lytB* gene, encoding HDR, grew slowly with depletion of photosynthetic pigments and slow lysis of the cells, but growth of the culture could be restored by supplementation with isopentenol and dimethylallyl alcohol, the nonphosphorylated alcohol counterparts of IDP and DMADP respectively. The authors also suggested that the IDP/DMADP synthase could potentially regulate the *in vivo* concentration and partitioning of IDP and DMADP [24]. Availability and isomerization of both IDP and DMADP is stringently controlled by the key enzyme isopentenyl diphosphate isomerase (IDP isomerase/IDI) in a reversible isomerization process. IDP isomerases are classified in two subfamilies: type I and type II with clearly distinct characteristics (for detail structure, features, biosynthesis and enzymatic activity see review [25].

Biosynthesis of all terpenoids begins with one or both of the C5 building blocks, IDP and DMADP [26] which are the products of the MEP pathway (Figure 1). These monomers are combined enzymatically to generate polymers through chain elongation reactions. With one molecule of each of IDP and DMADP, the enzyme geranyl diphosphate synthase (GDPS) generates GDP, a 10-carbon allylic diphosphate compound. GDP in turn serves as a precursor for formation of various monoterpenoids, and can undergo addition of another molecule of IDP to form farnesyl diphosphate (FDP, C15) through the action of FDP synthase (FDPS). FDP is the central precursor for sterols, triterpenoids, sesquiterpenoids and for the prenyl groups used for decoration of C15 prenylated proteins. Addition of another molecule of IDP to FDP generates geranylgeranyl diphosphate (GGDP, C20), a reaction catalyzed by the enzyme GGDP synthase (GGDPS). GGDP is the main branch point for diterpenoids, chlorophylls, carotenoids and the prenyl groups of C20 prenylated proteins.

Thus, GDP, FDP, and GGDP are the starting points for subsequent synthesis of terpenoid end products. Structural rearrangement of the carbon skeletons occurs through chain elongation, cyclization, isomerization and branching, by folding of the substrate and changes in the chemical bonding, to form any individual terpenoid structure [27]. The specific structure of terpenoids found in an organism define their functional role for growth and development of the organism, or for crucial ecological roles related to their existence in the natural habitats [28,29]. Formation of specific terpenoids requires terpene synthases (TPSs) using DMADP, GDP, FDP, or GGDP as the substrate. A recent review by Davies, Jinkerson and Posewitz [30] has discussed the product formation by TPSs in detail, and in particular implications for photoautotrophic growth. Most TPSs catalyze the formation of carbocation intermediates with multiple possible rearrangements of the carbon backbone that leads to multiple products from a single substrate. Often, a certain terpenoid cannot be formed in one single step, and special modifications for structural rearrangements are usually needed. Most of these modifications in terpenoid biosynthesis are performed by a class of heme-containing enzymes called cytochrome P450 monooxygenases (CP450) [31]. Several CP450s from plants and cyanobacteria have been identified as involved in terpenoid biosynthesis [32,33,34].

Genes, genetic regulation, enzyme structures and the enzyme activity in the MEP pathway has been described in higher plants, green algae, bacteria and cyanobacteria [10,17,35,36,37]. Metabolic regulation of the MEP pathway, including inputs of carbon, ATP, and reducing power was described in a recent review by Banerjee and Sharkey [21]. In plants, the MEP pathway is localized to the plastids [38]. As plant plastids originated from cyanobacteria (see review [39]), some genes of the MEP pathway are believed to have been brought into the eukaryotic cell by the cyanobacterial symbiont at the origin of the chloroplasts [40].

## 3. Terpenoids Produced in Cyanobacteria

Cyanobacteria are one of the oldest groups of organisms on the earth, with a fossil record of cyanobacterial-like organisms stretching back 3.3 to 3.5 billion years ago [41]. Cyanobacteria are Gram-negative photoautotrophic prokaryotes and are capable of performing oxygenic photosynthesis. They are the only group of organisms that are able to produce oxygen, reduce carbon dioxide and fix nitrogen in aerobic conditions, and thus they play a significant role in the nitrogen and carbon cycles [42]. Due to their ubiquitous occurrence in diverse natural habitats, cyanobacteria are able to produce a variety of secondary metabolites to adapt to the environmental conditions they may encounter. Terpenoids are a major group of secondary metabolites in many organisms including cyanobacteria.

Genes encoding each of the enzymes involved in the MEP pathway can be found in the complete genome sequence of *Synechocystis* [43] (Table 1), and also in the sequenced genomes of other cyanobacteria. The existence of a terpenoid biosynthesis pathway was also confirmed in *Synechocystis* sp. PCC 6714 through ^13^C-labelling studies [44], with results indicating that terpenoids are exclusively formed by MEP pathway in this organism.

The identification of all the genes required for the MEP pathway in cyanobacteria leads to the prediction that the pathway works similar to that in *E. coli*. However, the MEP pathway in photosynthetic organisms is directly linked to cellular photosynthetic activity as it uses pyruvate and glyceraldehyde3-phosphate as substrates, and energy supplied in the form of NADPH, reduced ferredoxin, CTP, and ATP, all of which are derived from photosynthesis. It has been shown for the cyanobacterium *T. elongatus* BP-1 that the formation of HMBDP from MEcDP catalyzed by GcpE (HDS, an Fe–S cluster-containing enzyme) is dependent on reduced ferredoxin for its activity [23].

**Table 1:** Enzymes and genes involved in the proposed *Synechocystis* MEP pathway *.

Enzymes	Genes	Alternative Gene Symbols	Gene ID	Gene Size (bp)
1-deoxy-d-xylulose 5-phosphate synthase (DXS)	*dxs*	-	*sll1945*	1923
1-deoxy-d-xylulose 5-phosphate reductoisomerase (DXR)	*dxr*	-	*sll0019*	1185
2-*C-*methyl-d-erythritol 4-phosphate cytidylyltransferase/CDP-ME synthase (MCT/CMS)	*ispD*	-	*slr0951*	693
4-(cytidine 5'-diphospho)-2-*C-*methyl-d-erythritol kinase (CMK)	*ispE*	-	*sll0711*	948
2-*C*-methyl-d-erythritol 2,4-cyclodiphosphate synthase (MDS/MCS)	*ispF*	*ygbB*	*slr1542*	486
4-hydroxy-3-methylbut-2-enyl diphosphate synthase (HDS)	*ispG*	*gcpE*	*slr2136*	1212
4-hydroxy-3-methylbut-2-enyl diphosphate reductase/IDP/DMADP synthase (HDR/IDS)	*ispH*	*lytB*	*slr0348*	1221
Isopentenyl diphosphate isomerase (IDI/IDI-2)	*idi*	*fni*	*sll1556*	1050
Geranyl diphosphate synthase (GDPS)/Farnesyl diphosphate synthase (FDPS)/Geranylgeranyl diphosphate synthase (GGDPS)	*crtE*	-	*slr0739*	909

* Enzymes and gene annotations as per data available from KEGG (http://www.genome.jp/kegg-bin/show_organism?menu_type=pathway_maps&org=syn) and CyanoBase (http://genome.microbedb.jp/cyanobase/Synechocystis [43]).

It is generally expected that interconversion of IDP and DMADP requires an IDP isomerase to further build all possible terpenoids from the basic structure of C5 as discussed earlier. Within the *Synechocystis* genome there are no open reading frames corresponding to any known type I IDP isomerase, as the one present in *E. coli* [45]. Searching for another alternative isomerase in this strain, Poliquin *et al.* [46] found the gene *sll1556* that showed sequence similarity to the type II IDP isomerase (IDP/DMADP isomerase or IDI-2) in *Salmonella enterica* [47]. By complementation studies with *Salmonella enterica* serovar Typhimurium strain RMC29 it was confirmed that ORF *sll1556* encodes a functional type II IDP isomerase [48] (Figure 1). After characterization of IDI-2 in *Synechocystis*, it was found that all sequenced cyanobacteria, proteobacteria and gram-positive bacteria possess this enzyme rather than a type I IDP isomerase [49]. In addition, it was shown that inactivation of *sll1556* in *Synechocystis* resulted in impaired terpenoids formation *in vitro* and *in vivo*, also affecting growth under high light conditions [46,48,50].

In a series of studies, it was reported that in extracts of *Synechocystis* cells grown under photosynthetic conditions, *in vitro* formation of products of the MEP pathway was not stimulated by pyruvate and GAP, but instead supply of intermediates originating from the pentose phosphate cycle (PPC) resulted in higher MEP pathway activity [46,51]. In addition, formidomycin, an inhibitor of DXR, affected neither the phototrophic growth of the *Synechocystis*, nor the terpenoids synthesis *in vitro* [51]. These results were taken to suggest that cyanobacteria might be utilizing intermediates of the PPC for terpenoids biosynthesis, rather than depending on GAP and pyruvate as substrates. However, a recent finding indicates that while addition of various PPC compounds supported *in vitro* terpenoids synthesis, the PPC compounds did not directly serve as substrates. From these results, the authors concluded that the *in vitro* stimulation by PPC compounds is indirect and does not occur via the MEP pathway [52].

In cyanobacteria, naturally occurring terpenoids fulfill roles similar to those in plants. However, the amount of terpenoids that can be isolated from plants or cyanobacteria are typically low, and extraction of the compounds is energy demanding. In addition, due to the structural complexity of many terpenoids, chemical synthesis is usually difficult, and costly, if at all possible [27]. An alternative and innovative approach to increase the terpenoid production is possible through metabolic engineering and synthetic biology [53,54].

By applying these techniques, native or heterologously expressed enzymes and pathways can be optimized to produce valuable terpenoids. In the last decade, microbial systems have been the focus of studies employing genetic manipulation and optimization of microbial metabolism to produce terpenoids for drugs and biofuels. Plant-derived pathways for terpenoid biosynthesis have been introduced through genetic engineering in microbial system particularly in the model organisms *E. coli* bacteria and in the yeast *Saccharomyces cerevisiae* [55,56,57] and recently in a few cyanobacteria (discussed below). Microbial systems have an advantage due to the ease with which they can be engineered through genetic manipulation to increase production yields. They are also compatible with large-scale fermentation processes, exhibit fast growth rates and easy extraction of products compared to the native plant systems. However, photosynthetic microorganisms like microalgae and cyanobacteria offer a further sustainable advantage in production of valuable compounds over both plants and other microbial systems. They are able, like plants, to directly utilize CO_2_ as their carbon source and light as their source of energy, and they do so more effectively with faster growth rates and better solar energy conversion than plants. At the same time, certain strains of cyanobacteria and microalgae have the same advantages as other microbial systems, such as being easily genetically modified, and can grow to high densities in photobioreactors, and offer simpler, more efficient extraction and purification procedures for the target molecule than plant systems [42,58]. Another important factor is the possibility of functional expression of enzymes and metabolic pathways from a plant system in the photosynthetic cyanobacterial cells rather than in another microbial system [59]. One example is the successful demonstration of *in vitro* and *in vivo* activities of CP450 enzymes from the endoplasmic reticulum membranes of *Sorghum bicolor* in the thylakoid membrane of *Synechococcus* sp. PCC 7002 [60]. It has been suggested that CP450 enzymes can be expressed in much larger amounts in the highly abundant thylakoids of a cyanobacterium compared to the endoplasmic reticulum [61] which makes heterologous expression in cyanobacteria a potentially more favorable option than expression in other microbial hosts. Furthermore, as described above, many terpenoid synthesis enzymes are dependent on the availability of reducing power in the form of NADPH or reduced ferredoxin, which can be expected to be more abundant in a photosynthetic cyanobacterial host than in another microbial system.

Through synthetic biology it may thus be possible to develop many variations of light-driven biosynthesis particularly of valuable terpenoids in cyanobacteria. Here we will discuss the current status of natural and heterologous production of terpenoids in cyanobacteria.

### 3.1. Hemiterpenes (C5)

The smallest terpenoids are those known as hemiterpenes, which are made up of a single terpenoids unit with five carbon atoms. One such compound, the volatile hydrocarbon isoprene (2-methyl-1,3-butadiene) is a valuable polymer building block in the synthetic chemistry industry and a potential biofuel. Currently, isoprene is used to manufacture products ranging from synthetic rubber to adhesives and perfumes. Terpenoid production from renewable materials as a substitute for petroleum-based products is regarded as an alternative solution to meet an increasing global demand for fuels and synthetic chemistry feedstock, in order to reduce the environmental impact of current production methods and ensure a sustained availability [62].

Various plant species has been found to possess the enzyme isoprene synthase, which catalyzes the conversion of DMADP to produce isoprene. Induction of production and release of isoprene to the surrounding environment from plants occurs under heat-stress conditions [63,64,65]. However, plants are unsuitable as production systems for renewable isoprene due to difficulty in harvesting a volatile compound from plants. Photosynthetic microorganisms provide a distinct advantage, as volatile compounds like isoprene can be harvested from the gas phase of a photosynthetic culture growing in a closed bioreactor [66].

Isoprene production has been reported to occur naturally in some marine cyanobacteria. In *Prochlorococcus* and *Synechococcus*, isoprene production was found to be influenced by light intensity and temperature of the environment they are grown in [67,68]. In another study, isoprene emission rates were reported from *Synechococcus* and *Trichodesmium* to depend on light intensity, cell volume and carbon content of the cells [69].

#### Heterologous Expression of Hemiterpenes in Cyanobacteria

Most cyanobacteria lack an isoprene synthase gene for isoprene formation. With the aim of producing isoprene for use as a renewable biofuel or feedstock in the synthetic chemistry industry, Lindberg *et al*. [70] heterologously expressed isoprene synthase (*IspS*) gene from *Pueraria montana* (kudzu) plant in *Synechocystis*. The resulting engineered strain was able to generate isoprene continuously along with the cyanobacterial growth. Additionally, an improved method for generating, sequestering, and trapping isoprene was also developed utilizing a sealed gaseous/aqueous two-phase photobioreactor [71].

To further improve the production and accumulation of isoprene from cyanobacteria, Bentley *et al*. [72] introduced non-native mevalonic acid (MVA) pathway genes from *Enterococcus faecalis* and *Streptococcus pneumoniae* (bacteria possessing the MVA pathway) in *Synechocystis*. The authors reported successful heterologous expression and accumulation of each protein from MEV pathway in the modified *Synechocystis*, and an approximately 2.5-fold increase of isoprene production compared to the strain expressing the isoprene synthase gene alone (Table 2). This study provided the first example of heterologous expression of an entire biosynthetic pathway in a photosynthetic microorganism.

### 3.2. Monoterpenes (C10)

Monoterpenes are the largest group of secondary metabolites of plants [73]. They contain 10 carbon atoms and are derived from condensation of IDP and DMADP in either head-to-tail or non-head-to-tail manner, or through condensation of two DMADP monomers. These compounds are important components of plant extracts and essential oils, possess high diversity and are widely used in pharmaceutical, cosmetic, agricultural and food industries [74]. In addition, monoterpenes may serve as a supplement to gasoline, and dimerization of the monoterpene units may generate second order fuel molecules that could be suitable for supplementing diesel type fuels.

Monoterpenes, due to their small molecular weight, are usually emitted as volatiles, either as single compound or in a combination of components. Like isoprene, they are released from plants [65]. In 2008 [75], it was reported that monoterpene emissions occurs at low rates from the marine cyanobacteria *Trichodesmium* IMS101 and *Synechococcus* RCC40. The function of emission of monoterpenes from cyanobacteria is still unknown, and further physiological and biochemical evidence is needed to understand this process. However, it is known that cyanobacteria perform many types of reactions known as biotransformations. In these processes, cell cultures or the endogenous enzymes have an ability to transform exogenous substrates into different products. Biotransformation enables obtaining novel or known compounds with higher yield, with lower cost [76,77,78]. Formation of monoterpenes through biotransformation has been reported from different types of cyanobacteria [79,80,81,82,83] and could potentially be utilized further for production of fuel and feedstock for the synthetic chemistry, or of compounds of interest for the pharmaceutical and cosmetics industries, depending on the specific compound formation and the properties of the products.

Some filamentous cyanobacterial species produce the volatile metabolite 2-methylisaborneol (2-MIB), which has no biological function known yet, but is known to give a taste and odor to water [84]. Interestingly enough, all cyanobacteria genomes sequenced to date are lacking genes homologous to known 2-MIB-synthesis genes from other organisms. However, through genome walking and using PCR methods, two genes were recognized in *Pseudanabaena* sp. and *Planktothricoids raciborskii*. The biosynthesis of 2-MIB has been characterized in strains of actinomycetes [85], and as was found in those organisms, production of 2-MIB in cyanobacteria occurs through a two-step reaction by methylation of GDP by methyl transferase (GDPMT), followed by cyclization of methyl-GDP by MIB synthase (MIBS) [86,87]. After comparison of the gene arrangement and functional sites between cyanobacteria and other organisms it was proposed that gene recombination and gene transfer probably occurred during the evolution of 2-MIB-associated genes that gives cyanobacteria a unique evolutionary lineage for the transformation of GDP to produce 2-MIB [87]. Light, temperature and nutrient level have been identified as major factors affecting levels of 2-MIB production [88,89]; however, other environmental factors might also be important for understanding the mechanism of expression of 2-MIB in cyanobacteria. A photopigment-dependent regulation of MIB synthesis and accumulation with increased cell metabolism was suggested for *Pseudanabaena articulate* and *Oscillatoria perornata* Skuja [90]. Very recently, an interesting experiment concluded that changes in levels of 2-MIB production depending on temperature are influenced by gene-level regulation, changes in central metabolic pathways, and increased cell growth [91].

#### Heterologous Expression of Monoterpenes in Cyanobacteria

Many enzymes that are needed for formation of different monoterpenes in plants are absent in cyanobacteria. Limonene is an example of a plant monoterpene which may serve as precursor to a range of commercially valuable products with a variety of applications [92], and which is not produced natively in cyanobacteria. Recently, heterologous expression of limonene synthase (LimS), the enzyme that catalyzes the final transformation of GDP to limonene, was demonstrated in cyanobacteria and production of limonene was observed. There are now several studies on limonene production in cyanobacteria, utilizing *limS*-genes from several different plant sources (*Schizonepeta tenuifolia*, *Sitka spruce*, *Mentha spicata*), which have been codon-optimized and heterologously expressed in the cyanobacteria *Synechocystis*, *Anabaena* sp. PCC 7120 and *Synechococcus* sp. PCC 7002 respectively [93,94,95] (Table 2). All these engineered cyanobacteria were shown to be successfully producing limonene.

β-phellandrene is another plant monoterpene, present in the essential oils of lavender and grand fir, and which is found on the surface of leaves and flowers where it may serve as part of a chemical defense against herbivores. β-phellandrene is used as a valuable ingredient in medicine, in cosmetics, and in perfumes, and has also been proposed as a candidate compound to be developed as a renewable fuel [96]. β-phellandrene is a product of the plant chloroplast localized MEP pathway and is directly synthesized from GDP by β-phellandrene synthase (β-PHLS). Cyanobacteria lack β-PHLS and cannot synthesize β-phellandrene naturally. To produce this monoterpene from cyanobacteria, a codon-optimized β-PHLS gene from *Lavandula angustifolia* (lavender) was heterologously expressed in *Synechocystis* [96] (Table 2). Secretion of β-phellandrene was observed from the transgenic cells, and was easily separated from the liquid culture. This system gives the advantage of separation of the product from the biomass, without affecting cellular growth, and enables a continuous production process. Recently, another study reported improvement of the system by employing different promoters to drive expression of the PHLS-gene, and the authors concluded that heterologous expression of the PHLS protein is the rate-limiting step in photosynthetic β-phellandrene production in cyanobacteria [97].

The monoterpene alcohol linalool is a known component of many fragrant herbs, and contributes to the unique pleasant smell of the lavender plant. Linalool has several valuable biological properties, recently reviewed by Aprotosoaie *et al.* [98]. As for isoprene and phellandrene, linalool also has a high energy density that could make it suitable for replacement of petroleum fuels. Linalool is naturally produced from the MEP pathway of certain plants, and is synthesized by linalool synthase (LinS) from GDP [99]. Cyanobacteria generally lack a gene encoding LinS. With the aim to produce linalool from cyanobacteria, a linalool synthase gene from Norway Spruce was introduced to *Anabaena* sp. PCC 7120 [100]. Over-expression and secretion of linalool using CO_2_ as the carbon source was reported from the engineered cyanobacteria (Table 2).

**Table 2:** Heterologous production of terpenoids in cyanobacteria.

Host Strain	Terpenoids Produced	Gene and Source	Promoter	Maximum Amount Produced	Reference
*Synechocystis*	Isoprene	*Synechocystis* codon optimized isoprene synthase (*IspS*) from *Pueraria montana* (kudzu)	*psbA2*	50 μg g^−1^ DCW	[70]
*Synechocystis*	Isoprene	*Synechocystis* codon optimized isoprene synthase (*IspS*) from *Pueraria montana* (kudzu)	*psbA2*	150 μg L ^−1^	[71]
*Synechocystis*	Isoprene	MVA pathway genes encoding the enzymes, Hmg-CoA synthase (HmgS) and Hmg-CoA reductase (HmgR) from *Enterococcus faecalis*;	*psbA2*	250 μg g^−1^ DCW	[72]
		Acetyl-CoA acetyl transferase (AtoB) from *E. coli*;			
		IDP isomerase (Fni), mevalonic acid kinase (MK), mevalonic acid 5-diphoshate decarboxylase (PMD), and mevalonic acid 5-phosphate kinase (PMK) from *Streptococcus pneumoniae*			
*Synechocystis*	Limonene	*Synechocystis* codon optimized limonene synthase (*LimS*) from *Schizonepeta tenuifolia*, and overexpression with native genes *dxs*, *crtE* and *idi*	*Ptrc*	41 µg L ^−1^ day^−1^, 56 µg L ^−1^ day^−1^	[93]
*Synechococcus* sp. PCC 7002	Limonene	*Synechococcus* codon optimized L-limonene synthase from *Mentha spicata*	*cpcBA* (*cpc*)	4 mg L^−1^	[95]
*Anabaena* sp. PCC 7120	Limonene	Limonene synthase (*LimS*) from *Picea sitchensis* (Sitka spruce), Synthetic DXP operon with *dxs* from *E. coli*; IDI from *Haematococcus pluvialis*; and GDPS from *Mycoplasma tuberculosis* for co-expression with *LimS*	dual promoter *Pnir*/*PpsbA1*	3.6 ± 0.5 μg L^−1^ OD^−1^ h^−1^	[94]
*Synechocystis*	β-phellandrene	*Synechocystis* codon-optimized β-phellandrene synthase (β-PHLS) gene from *Lavandula angustifolia* (lavender)	*psbA2*	1.0 μg L^−1^ h^−1^	[96]
*Synechocystis*	β-phellandrene	*Synechocystis* codon-optimized β-phellandrene synthase (β-PHLS) gene from *Lavandula angustifolia* (lavender)	*psbA2*, *psbA2* (no AT -box), *ptrc-*T7-g10 and *cpc*	253.8 ± 54.8 μg g^−1^ DCW	[97]
*Anabaena* sp. PCC 7120	Linalool	Linalool synthase (LinS) from *Picea abies* (Norway Spruce)			[100], Patent
*Synechocystis*	β-caryophyllene	β-caryophyllene synthase gene (QHS1) from *Artemisia annua*	*psbA2*	3.7 μg g^−1^ DCW week^−1^	[101]
*Anabaena* sp. PCC 7120	Farnesene	*Anabaena* codon-optimized farnesene synthesize (FaS) gene from *Picea abies* (Norway Spruce)	dual promoter *Pnir*/*PpsbA1*	305.4 ± 17.7 μg·L^−1^	[102]
*Synechococcus* sp. PCC 7002	Bisabolene	*Synechococcus* codon optimized (*E*)-a-bisabolene synthase from *Abies grandis* (Grand Fir)	*cpcBA* (*cpc*)	0.6 mg L^−1^	[95]

### 3.3. Sesquiterpenes (C15)

Sesquiterpenes, with 15 carbons, are derived from the condensation of one additional IDP monomer to the C10 monoterpene GDP to form farnesyl diphosphate, FDP. A large number of sesquiterpene compounds can be produced, due to the increased variability made possible by the increased chain length, and sesquiterpenes are found to contribute to a wide range of biological functions [103].

The sesquiterpene-derived alcohol known as geosmin is produced from several organisms including cyanobacteria. Geosmin gives an earthy taste and musty odor in drinking water supplies, and cyanobacteria were reported to be largely responsible for this [84]. The biochemical mechanisms of geosmin synthesis have been examined in a model cyanobacterium, *Nostoc punctiforme* PCC 73102 [104]. In concurrence with previous reports regarding geosmin synthesis in actinomycetes, formation of geosmin in cyanobacteria results from conversion of the sesquiterpene precursor FDP through two steps catalyzed by a bi-functional geosmin synthase, encoded by the *geoA* gene, in the presence of Mg^2+^, as confirmed by *in vitro* experiments [104]. In another study, the same geosmin synthase was identified from *Nostoc punctiforme* PCC 73102 [32]. However, upon expression of the enzyme in *E. coli*, the authors of this study could not detect geosmin production, but synthesis of germacradienol, germacrene D, and germacrene A, all known alternative products of geosmin synthases, was observed. Furthermore, the authors identified a sesquiterpene synthase present in both *Anabaena* sp. PCC 7120 and *Anabaena variabilis* ATCC 29413, whose activity in *E. coli* as well as in *in vitro* assays resulted in formation of germacrene A, and an additional sesquiterpene in *Nostoc punctiforme* from which synthesis of 8a-*epi*-α-selinene was observed. The genes encoding these two latter sesquiterpene synthases were found to be located in a gene cluster together with cytochrome CP450 enzymes. However, their functional expression depending on different environmental conditions still needs to be investigated in order to understand their function in the organism. In a study examining sesquiterpene formation in the filamentous cyanobacterium *Calothrix* PCC 7507, geosmin was found to be the dominating compound. Further sesquiterpenes were detected, including germacrene D, isodihydroagarofuran, 6,11-epoxyisodaucane and eremophilone, which was reported to be toxic to certain specific invertebrates [105].

#### Heterologous Expression of Sesquiterpenes in Cyanobacteria

β-caryophyllene is a bicyclic sesquiterpene compound found in many plants, including spices such as black pepper [106] and cloves [107], and also in the essential oil of *Cannabis sativa* [108]. Traditionally, this compound has been used in the cosmetics industry to provide a woody, spicy aroma to cosmetics and perfumes. It has also been found to be potentially effective as an anesthetic [107] and as an anti-inflammatory agent, probably acting through binding of a cannabinoid receptor [109]. There are no reports of cyanobacteria producing β-caryophyllene naturally, and in the model cyanobacterium *Synechocystis* there are no known genes for its synthesis. *In vivo* production of the β-caryophyllene in *Synechocystis* was reported for the first time after stable insertion of β-caryophyllene synthase gene (QHS1) from *Artemisia annua* into the genome of the *Synechocystis* [101].

Farnesene is a sesquiterpene directly derived from FDP which is used as lubricant, in cosmetics, in fragrances and also as a biofuel [55]. Production of farnesene is of interest since it is considered to be an attractive biofuel, for its volatile nature and high energy density. In a recent study, the filamentous, heterocystous cyanobacterium *Anabaena* sp. PCC 7120 was used as host organism for expression of a codon-optimized farnesene synthetase (FaS) gene from Norway spruce, to produce and secrete farnesene [102]. The engineered strain showed higher photosynthetic activity with increasing light intensities and the authors suggest that farnesene could possibility function as an added carbon sink in the cyanobacterium, thereby influencing the activity of photosynthesis.

The enzyme α-bisabolene synthase catalyzes formation of α-bisabolene in a wide range of plants. In a study on cyanobacterial production of limonene and bisabolene, heterologous expression of an (*E*)-α-bisabolene synthase gene from *Abies grandis* in *Synechococcus* sp. PCC 7002 resulted in formation of α-bisabolene in this cyanobacterium [95]. However, the rate of production was lower than that obtained for limonene. The authors concluded that since cyanobacteria in general do not produce large amounts of sesquiterpenes, a low availability of the FDP precursor may be limiting also for heterologous production of sesquiterpenes.

### 3.4. Diterpenes (C20)

Diterpenoids are structurally diverse C20 hydrocarbons derived from addition of one molecule of IDP to FDP to form geranyl geranyl diphosphate, GGDP. From GGDP, structural diversity is created through the action of two enzyme families, the diterpene synthases and cytochrome CP450 [61]. Due to their structural complexity, chemical synthesis of these compounds is typically difficult, and availability of natural diterpenoids is generally limited since laborious extraction procedures are necessary to isolate them from the original plant sources. It is, therefore, interesting to find microbial hosts to produce these compounds, and cyanobacteria may provide a suitable environment for the necessary enzymes, as they naturally resemble the conditions in chloroplasts.

Naturally cyanobacteria do produce several diterpenes with specific functions, e.g., tolypodiol, a diterpenoid compound detected from *Tolypothrix nodosa* which was shown to possess anti-inflammatory properties [110]. A novel extracellular metabolite “noscomin” with an uncommon diterpenoid skeleton was reported from *Nostoc commune* strain [111,112] and was found to have antibacterial properties. Two abietane diterpenes isolated from the cyanobacterium *Microcoleous lacustris* showed antibacterial activity against few specific bacteria [113].

GGDP is also the substrate for formation of phytyl, by the action of the enzyme geranylgeranyl reductase. Phytyl is used in plants and cyanobacteria for formation of tocopherols, and serves as substrate for adding the phytol side chains of chlorophyll, ubiquinones, plastoquinone and phylloquinone. Inactivation of the gene encoding geranylgeranyl reductase, *chlP*, in *Synechocystis* resulted in formation of geranylgeranylated chlorophyll *a* instead of the normal phytylated form, formation of α-tocotrienol instead of α-tocopherol, and the mutant strain was found to be incapable of sustaining photoautotrophic growth [114].

### 3.5. Triterpenes (C30)

Triterpenes are 30-carbon hydrocarbons, biosynthesized through the head-to-head condensation of two molecules of FDP to form squalene. Squalene in turn serves as the precursor for formation of triterpenoids including the bacterial hopanoids and eukaryotic sterols. Squalene itself is a natural antioxidant and has commercial use in cosmetics, nutrition and in vaccines [115,116,117], and is extracted for commercial use from shark liver oil, or olive or other plant oils.

In cyanobacteria, some but not all strains possess a squalene synthase gene, *sqs*. Functional characterization of a squalene synthase in cyanobacteria was first reported in *T. elongates* BP-1 [118]. Recently, an *sqs* gene was also identified and its function experimentally verified by inactivation in *Synechocystis* [119]. In the same study, it was shown that inactivation of a gene putatively encoding a squalene hopene cyclase, *shc*, causes squalene to accumulate in *Synechocystis* cells. Shc is the enzyme responsible for cyclization of squalene to form hopene, the precursor for formation of the family of triterpene compounds known as hopanoids [120,121].

Hopanoids, pentacyclic triterpenoid compounds found in many bacteria, have been used as biological markers, e.g., presence of certain distribution of hopanoids in rocks and oil deposits provides information about environmental conditions during deposition as well as the age of the sample [122]. In bacteria, hopanoids are formed as building blocks for membrane biogenesis and have a role in maintaining membrane integrity and permeability [123,124], as wells as in coping with external stress, e.g., oxygen diffusion [125], pH stress [126], antimicrobial resistance [127] and ethanol stress [128,129]. Recently, the hopanoid biosynthetic pathway has been characterized in some gram-negative bacteria [130,131]. A wide variety of hopanoid structures has been reported from different organisms, suggesting that there are specific functions which are yet to be discovered. The role of hopanoids in cyanobacteria is not well studied yet. Nevertheless, presence of several hopanoid compounds has been reported from cyanobacteria [132,133,134,135] and it has been suggested that they might play a role in protecting against extreme environmental conditions [134].

### 3.6. Tetraterpenes (C40)

Tetraterpenes with 40 carbons are derived from phytoene formed by two C20 GGDP in a head-to-head condensation reaction [136]. One group of tetraterpenes is the carotenoids pigments, which is a family of compounds with a large structural diversity. Carotenoids have important biological functions, with roles in light capture, antioxidative activity and protection from the damaging effects of free radicals, synthesis of plant hormones, and as structural components in membranes. Apart from their biological functions, these pigments have commercial use as food colorants, animal food supplement, in nutraceuticals and pharmaceuticals. Carotenoids are synthesized both by photosynthetic and nonphotosynthetic organisms, however, in photosynthetic organisms, including cyanobacteria, they are essential components in photosynthetic membranes and together with chlorophyll they stabilize photosynthetic reaction centers and protect and regulate oxygenic photosynthesis [137,138,139]. The topic of carotenoids is too vast to be covered here, and has been well described previously. For reviews on carotenoids, their synthesis, and cellular functions, see [137,140,141,142].

## 4. Strategies to Enhance Terpenoid Production

Production of terpenoids is subject to regulation in the cell, and the maximum productivity is determined by rate-limiting steps in the MEP pathway. Several attempts have been made in recent studies to alleviate the limitations on productivity of terpenoids in cyanobacterial model organisms. DXS, the enzyme catalyzing the first step of the MEP pathway, has been identified as a bottleneck of the pathway [143]. To direct the carbon flux towards product formation, Kudoh *et al.* [144] developed a DXS overexpression strain in *Synechocystis*, by expressing an extra copy of the native *Synechocystis* *dxs* gene under control of the highly active *psbA2* promoter. The extra expression of DXS in the modified strain lead to an increase in carotenoid levels, by a factor of about 1.5, and a decrease in glycogen levels, which lead the authors to suggest that the increase in carotenoid content was due to consumption of glycogen. It was concluded from the results that through optimization of gene expression and restricted synthesis of storage compounds, an improved terpenoid product yield is possible in cyanobacteria. However, in another study in *Synechococcus* sp. PCC 7002, there was no increase in terpenoid product formation when glycogen synthesis was inactivated. Rather, secretion of central metabolites not connected to terpenoid biosynthesis was observed [95].

In another recent study, overexpression of three native enzymes, DXS, IDI and GDPS, in *Synechocystis* under the control of the strong *trc* promoter, lead to an increase in formation of limonene (formed by heterologous expression of *limS*, see further above and Table 2) by a factor 1.4, similar to the effect on carotenoid formation seen in the study by [143] by expression of *dxs* alone [93]. In a similar study on a limonene producing strain of *Anabaena* sp. PCC 7120, the authors attempted to increase flux through the MEP pathway by heterologous overexpression of DXS from *E. coli*, IDI from *Haematococcus pluvalis*, and GDPS from *Mycoplasma tuberculosis* in an operon together with *limS*, under control of a double P*nir*-P*psbA1* promoter construct [94]. This lead to an increase in formation of limonene by a factor of 2.3 in low light, but production increased by a factor of 6.8 under high light. Furthermore, it was observed that the limonene producing strains used in the study exhibited higher oxygen evolution under high light than did the wild type strain, indicating that limonene production acted as an extra carbon sink in the cells. These results, compared to the smaller effects on product yield observed in the studies on overexpression of native MEP pathway genes in *Synechocystis*, may indicate an advantage gained by using heterologous enzymes for enhancing carbon flux through the MEP pathway, as this may avoid native regulation of the enzymes interfering with their activity.

Taking this approach one step further, as mentioned above, Bentley *et al*. [72] heterologously expressed the entire MEV pathway, under control of the *psbA2* promoter, in an isoprene producing strain of *Synechocystis* resulting in a 2.5 times increased yield of isoprene. The increase in product formation observed in this study is due to the availability of additional substrate for the cell to direct to terpenoid formation, since the MEV pathway uses acetyl-CoA as substrate rather than GAP and pyruvate, but is likely limited by bottlenecks in the introduced pathway.

## 5. Future Perspective

Terpenoids are essential to all living organisms, and there are many aspects of their biosynthesis that remains to be elucidated. There is also likely to be a great diversity of terpenoid compounds that have still not been identified. As more information about biosynthesis of specific terpenoids becomes available, especially regarding the wide variety of terpenoid secondary metabolites with useful properties from plants, it also becomes interesting to establish microbial platforms for their production on larger scale. Cyanobacteria may turn out to be superior host organisms for heterologous production of terpenoids, since they do form relatively large amounts of these compounds naturally, mainly in the form of carotenoids and as the phytol side chain of chlorophyll, and since they provide a plant-like environment inside the cells, with access to reducing power derived from photosynthesis to drive the formation of terpenoids by the action of terpene synthases and cytochrome P450 enzymes. As described above, some non-native terpenoids have already been successfully produced in model cyanobacteria.

As biotechnological production organisms, cyanobacteria have the great advantage of being photosynthetic and thus able to grow on minimal nutrients with sunlight as their energy source. However, they are limited in their ability to produce terpenoids due to regulation and constraints on the MEP pathway. Attempts to alleviate those constraints have recently been made, as described above, but have so far only been moderately successful and further enhancements are necessary to achieve commercially viable production. To enhance target product formation, metabolic flux needs to be redirected for production of the specific terpenoid. For easy recovery of the product from the culture, it is helpful to focus on products that are either volatile or hydrophobic compounds that can be excreted from the cells and are easily separated from the growth medium. For products that are not excreted from the cells, introduction of specific engineered transporters can be considered as a way of achieving efficient export of the intracellularly synthesized terpenoid molecules to the medium.

There is in general for cyanobacteria a lack of appropriate tools for genetic engineering, and development of promoters, vectors, and regulatory systems to use in engineering of new and native pathways still needs to be further developed. Furthermore, as is made evident in this review, the native biosynthesis of terpenoids in cyanobacteria is far from fully investigated, and many questions remain about the regulation and control of the MEP pathway as well as synthesis of specific terpenoids. There is also likely to exist still unknown terpenoid compounds produced by cyanobacteria. Further investigation into the native terpenoid biosynthesis in cyanobacteria is vital, as it will provide knowledge necessary for successful engineering of these organisms for production of terpenoids of interest for pharmaceutical applications, in nutrition, or as future biofuels.

## References

[1] Mazid M., Khan T., Mohammad F. (2011). Role of secondary metabolites in defense mechanisms of plants. Biol. Med..

[2] Tholl D. (2006). Terpene synthases and the regulation, diversity and biological roles of terpene metabolism. Curr. Opin. Plant Biol..

[3] Van Agtmael M.A., Eggelte T.A., van Boxtel C.J. (1999). Artemisinin drugs in the treatment of malaria: From medicinal herb to registered medication. Trends Pharmacol. Sci..

[4] Slichenmyer W.J., von Hoff D.D. (1991). Taxol: A new and effective anti-cancer drug. Anticancer. Drugs.

[5] Haridas V., Higuchi M., Jayatilake G.S., Bailey D., Mujoo K., Blake M.E., Arntzen C.J., Gutterman J.U. (2001). Avicins: Triterpenoid saponins from *Acacia victoriae* (Bentham) induce apoptosis by mitochondrial perturbation. Proc. Natl. Acad. Sci. USA.

[6] Guzman M.L., Rossi R.M., Karnischky L., Li X., Peterson D.R., Howard D.S., Jordan C.T. (2005). The sesquiterpene lactone parthenolide induces apoptosis of human acute myelogenous leukemia stem and progenitor cells. Blood.

[7] Fujioka T., Kashiwada Y., Kilkuskie R.E., Cosentino L.M., Ballas L.M., Jiang J.B., Janzen W.P., Chen I.S., Lee K.H. (1994). Anti-AIDS agents, 11. Betulinic acid and platanic acid as anti-HIV principles from *Syzigium claviflorum*, and the anti-HIV activity of structurally related triterpenoids. J. Nat. Prod..

[8] Riccioni G. (2009). Carotenoids and cardiovascular disease. Curr. Atheroscler. Rep..

[9] Johnson E.J. (2002). The role of carotenoids in human health. Nutr. Clin. Care.

[10] Chang W.-C., Song H., Liu H.-W., Liu P. (2013). Current development in isoprenoid precursor biosynthesis and regulation. Curr. Opin. Chem. Biol..

[11] Rohmer M., Knani M., Simonin P., Sutter B., Sahm H. (1993). Isoprenoid biosynthesis in bacteria: A novel pathway for the early steps leading to isopentenyl diphosphate. Biochem. J..

[12] Rohmer M., Seemann M., Horbach S., Bringer-Meyer S., Sahm H. (1996). Glyceraldehyde 3-Phosphate and pyruvate as precursors of isoprenic units in an alternative non-mevalonate pathway for terpenoid biosynthesis. J. Am. Chem. Soc..

[13] Rohmer M. (1999). The discovery of a mevalonate-independent pathway for isoprenoid biosynthesis in bacteria, algae and higher plants. Nat. Prod. Rep..

[14] Rohmer M. (2003). Mevalonate-independent methylerythritol phosphate pathway for isoprenoid biosynthesis. Elucidation and distribution. Pure Appl. Chem..

[15] Lois L.M., Campos N., Putra S.R., Danielsen K., Rohmer M., Boronat A. (1998). Cloning and characterization of a gene from *Escherichia coli* encoding a transketolase-like enzyme that catalyzes the synthesis of d-1-deoxyxylulose 5-phosphate, a common precursor for isoprenoid thiamin, and pyridoxol biosynthesis. Proc. Natl. Acad. Sci. USA.

[16] Miller B., Heuser T., Zimmer W. (1999). A *Synechococcus leopoliensis* SAUG 1402–1 operon harboring the 1-deoxyxylulose 5-phosphate synthase gene and two additional open reading frames is functionally involved in the dimethylallyl diphosphate synthesis. FEBS Lett..

[17] Cordoba E., Salmi M., León P. (2009). Unravelling the regulatory mechanisms that modulate the MEP pathway in higher plants. J. Exp. Bot..

[18] Yin X., Proteau P.J. (2003). Characterization of native and histidine-tagged deoxyxylulose 5-phosphate reductoisomerase from the cyanobacterium *Synechocystis* sp. PCC6803. Biochim. Biophys. Acta.

[19] Thérisod M., Fischer J.-C., Estramareix B. (1981). The origin of the carbon chain in the thiazole moiety of thiamine in *Escherichia coli*: Incorporation of deuterated 1-deoxy-d-threo-2-pentulose. Biochem. Biophys. Res. Commun..

[20] Hill R.E., Himmeldirk K., Kennedy I.A., Pauloski R.M., Sayer B.G., Wolf E., Spenser I.D. (1996). The biogenetic anatomy of vitamin B_6_: A ^13^C-NMR investigation of the biosynthesis of pyridoxol in *Escherichia coli*. J. Biol. Chem..

[21] Banerjee A., Sharkey T.D. (2014). Methylerythritol 4-phosphate (MEP) pathway metabolic regulation. Nat. Prod. Rep..

[22] Seemann M., Tse Sum Bui B., Wolff M., Miginiac-Maslow M., Rohmer M. (2006). Isoprenoid biosynthesis in plant chloroplasts via the MEP pathway: Direct thylakoid/ferredoxin-dependent photoreduction of GcpE/IspG. FEBS Lett..

[23] Okada K., Hase T. (2005). Cyanobacterial non-mevalonate pathway: (*E*)-4-hydroxy-3-methylbut-2-enyl diphosphate synthase interacts with ferredoxin in *Thermosynechococcus elongatus* BP-1. J. Biol. Chem..

[24] Cunningham F.X., Lafond T.P., Gantt E. (2000). Evidence of a role for LytB in the nonmevalonate pathway of isoprenoid biosynthesis. J. Bacteriol..

[25] Berthelot K., Estevez Y., Deffieux A., Peruch F. (2012). Isopentenyl diphosphate isomerase: A checkpoint to isoprenoid biosynthesis. Biochimie.

[26] Sacchettini J.C., Poulter C.D. (1997). Creating isoprenoid diversity. Science.

[27] Ajikumar P.K., Tyo K., Carlsen S., Mucha O., Phon T.H., Stephanopoulos G. (2008). Terpenoids: Opportunities for biosynthesis of natural product drugs using engineered microorganisms. Mol. Pharm..

[28] Ramak P., Osaloo S.K., Sharifi M., Ebrahimzadeh H., Behmanesh M. (2014). Biosynthesis, regulation and properties of plant monoterpenoids. J. Med. Plant Res..

[29] Holopainen J.K., Himanen S.J., Yuan J.S., Chen F., Stewart C.N., Ramawat K.G., Mérillon J.-M. (2013). Ecological functions of terpenoids in changing climates. Natural Products.

[30] Davies F.K., Jinkerson R.E., Posewitz M.C. (2014). Toward a photosynthetic microbial platform for terpenoid engineering. Photosynth. Res..

[31] Werck-Reichhart D., Feyereisen R. (2000). Cytochromes P450: A success story. Genome Biol..

[32] Agger S.A., Lopez-Gallego F., Hoye T.R., Schmidt-Dannert C. (2008). Identification of sesquiterpene synthases from *Nostoc punctiforme* PCC 73102 and *Nostoc* sp. strain PCC 7120. J. Bacteriol..

[33] Robert F.O., Pandhal J., Wright P.C. (2010). Exploiting cyanobacterial P450 pathways. Curr. Opin. Microbiol..

[34] Zhao Y.-J., Cheng Q.-Q., Su P., Chen X., Wang X.-J., Gao W., Huang L.-Q. (2014). Research progress relating to the role of cytochrome P450 in the biosynthesis of terpenoids in medicinal plants. Appl. Microbiol. Biotechnol..

[35] Hunter W.N. (2007). The non-mevalonate pathway of isoprenoid precursor biosynthesis. J. Biol. Chem..

[36] Rohmer M., Timmis K.N. (2010). Hopanoids. Handbook of Hydrocarbon and Lipid Microbiology.

[37] Zhao L., Chang W.-C., Xiao Y., Liu H.-W., Liu P. (2013). Methylerythritol phosphate pathway of isoprenoid biosynthesis. Annu. Rev. Biochem..

[38] Rodríguez-Concepción M., Boronat A. (2002). Elucidation of the methylerythritol phosphate pathway for isoprenoid biosynthesis in bacteria and plastids. A metabolic milestone achieved through genomics. Plant Physiol..

[39] Ginger M.L., McFadden G.I., Michels P.A.M. (2010). Rewiring and regulation of cross-compartmentalized metabolism in protists. Philos. Trans. R. Soc..

[40] Lange B.M., Rujan T., Martin W., Croteau R. (2000). Isoprenoid biosynthesis: The evolution of two ancient and distinct pathways across genomes. Proc. Natl. Acad. Sci. USA.

[41] Schopf J.W., Packer M.B. (1987). Early Archean (3.3-billion to 3.5-billion-year-old) microfossils from Warrawoona Group, Australia. Science.

[42] Parmar A., Singh N.K., Pandey A., Gnansounou E., Madamwar D. (2011). Cyanobacteria and microalgae: A positive prospect for biofuels. Bioresour. Technol..

[43] Kaneko T., Tabata S. (1997). Complete genome structure of the unicellular cyanobacterium *Synechocystis* sp. PCC 6803. Plant Cell Physiol..

[44] Disch A., Schwender J., Müller C., Lichtenthaler H.K., Rohmer M. (1998). Distribution of the mevalonate and glyceraldehyde phosphate/pyruvate pathways for isoprenoid biosynthesis in unicellular algae and the cyanobacterium *Synechocystis* PCC 6714. Biochem. J..

[45] Ershov Y., Gantt R.R., Cunningham F.X., Gantt E. (2000). Isopentenyl diphosphate isomerase deficiency in *Synechocystis* sp. strain PCC 6803. FEBS Lett..

[46] Poliquin K., Ershov Y.V., Cunningham F.X.J., Woreta T.T., Gantt R.R., Gantt E. (2004). Inactivation of *sll1556* in *Synechocystis* Strain PCC 6803 impairs isoprenoid biosynthesis from pentose phosphate cycle substrates *in vitro*. J. Bacteriol..

[47] Kaneda K., Kuzuyama T., Takagi M., Hayakawa Y., Seto H. (2001). An unusual isopentenyl diphosphate isomerase found in the mevalonate pathway gene cluster from *Streptomyces* sp. strain CL190. Proc. Natl. Acad. Sci. USA.

[48] Barkley S.J., Desai S.B., Poulter C.D. (2004). Type II isopentenyl diphosphate isomerase from *Synechocystis* sp. strain PCC 6803. J. Bacteriol..

[49] Barkley S.J., Cornish R.M., Poulter C.D. (2004). Identification of an Archaeal type II isopentenyl diphosphate isomerase in *Methanothermobacter thermautotrophicus*. J. Bacteriol..

[50] Poliquin K., Cunningham F.X., MacDonald I., Gantt R.R., Gantt E., Allen J.F., Gantt E., Golbeck J., Osmond B. (2008). Impaired isoprenoid biosynthesis: A competitive disadvantage under light stress in *Synechocystis* PCC 6803. Photosynthesis. Energy from the Sun: 14th International Congress on Photosynthesis.

[51] Ershov Y.V., Gantt R.R., Cunningham F.X., Gantt E. (2002). Isoprenoid biosynthesis in *Synechocystis* sp. strain PCC 6803 is stimulated by compounds of the pentose phosphate cycle but not by pyruvate or deoxyxylulose-5-phosphate. J. Bacteriol..

[52] Poliquin K., Cunningham F.X., Gantt R.R., Gantt E., Bach T.J., Rohmer M. (2013). Interaction of isoprenoid pathway enzymes and indirect stimulation of isoprenoid biosynthesis by pentose phosphate cyacle substrates in *Synechocystis* PCC 6803. Isoprenoid Synthesis in Plants and Microorganisms: New Concepts and Experimental Approaches.

[53] Winter J.M., Tang Y. (2012). Synthetic biological approaches to natural product biosynthesis. Curr. Opin. Biotechnol..

[54] Kern A., Tilley E., Hunter I.S., Legisa M., Glieder A. (2007). Engineering primary metabolic pathways of industrial micro-organisms. J. Biotechnol..

[55] Buijs N.A., Siewers V., Nielsen J. (2013). Advanced biofuel production by the yeast *Saccharomyces cerevisiae*. Curr. Opin. Chem. Biol..

[56] Martin V.J.J., Pitera D.J., Withers S.T., Newman J.D., Keasling J.D. (2003). Engineering a mevalonate pathway in *Escherichia coli* for production of terpenoids. Nat. Biotechnol..

[57] Peralta-Yahya P.P., Zhang F., del Cardayre S.B., Keasling J.D. (2012). Microbial engineering for the production of advanced biofuels. Nature.

[58] Ducat D.C., Way J.C., Silver P.A. (2011). Engineering cyanobacteria to generate high-value products. Trends Biotechnol..

[59] Xue Y., Zhang Y., Grace S., He Q. (2014). Functional expression of an *Arabidopsis* p450 enzyme, p-coumarate-3-hydroxylase, in the cyanobacterium *Synechocystis* PCC 6803 for the biosynthesis of caffeic acid. J. Appl. Phycol..

[60] Lassen L.M., Nielsen A.Z., Olsen C.E., Bialek W., Jensen K., Møller B.L., Jensen P.E. (2014). Anchoring a plant cytochrome P450 via PsaM to the thylakoids in *Synechococcus* sp. PCC 7002: Evidence for light-driven biosynthesis. PLoS One.

[61] Jensen P.E., Lassen M.M., Gnanasekaran T., Nielsen A.Z., Møller B.L. Using synthetic biology to retarget biosynthetic pathways to the chloroplast for direct access to the products of photosynthesis. http://www.isb.vt.edu/news/2013/Jul/JLGNM.pdf.

[62] Zhao Y., Yang J., Qin B., Li Y., Sun Y., Su S., Xian M. (2011). Biosynthesis of isoprene in *Escherichia coli* via methylerythritol phosphate (MEP) pathway. Appl. Microbiol. Biotechnol..

[63] Singsaas E.L., Lerdau M., Winter K., Sharkey T.D. (1997). Isoprene increases thermotolerance of isoprene-emitting species. Plant Physiol..

[64] Sharkey T.D., Yeh S. (2001). Isoprene emission from plants. Annu. Rev. Plant Physiol. Plant Mol. Biol..

[65] Sharkey T.D., Wiberley A.E., Donohue A.R. (2008). Isoprene emission from plants: Why and how. Ann. Bot..

[66] Melis A. (2012). Photosynthesis-to-fuels: From sunlight to hydrogen, isoprene, and botryococcene production. Energy Environ. Sci..

[67] Shaw S.L., Chisholm S.W., Prinn R.G. (2003). Isoprene production by *Prochlorococcus*, a marine cyanobacterium, and other phytoplankton. Mar. Chem..

[68] Shaw S.L., Gantt B., Meskhidze N. (2010). Production and emissions of marine isoprene and monoterpenes: A Review. Adv. Meteorol..

[69] Bonsang B., Gros V., Peeken I., Yassaa N., Bluhm K., Zöllner E., Sarda-Esteve R., Williams J. (2010). Isoprene emission from phytoplankton monocultures: The relationship with chlorophyll-*a*, cell volume and carbon content. Environ. Chem..

[70] Lindberg P., Park S., Melis A. (2010). Engineering a platform for photosynthetic isoprene production in cyanobacteria, using *Synechocystis* as the model organism. Metab. Eng..

[71] Bentley F.K., Melis A. (2012). Diffusion-based process for carbon dioxide uptake and isoprene emission in gaseous/aqueous two-phase photobioreactors by photosynthetic microorganisms. Biotechnol. Bioeng..

[72] Bentley F.K., Zurbriggen A., Melis A. (2014). Heterologous expression of the mevalonic acid pathway in cyanobacteria enhances endogenous carbon partitioning to isoprene. Mol. Plant.

[73] Banthorpe D.V., Charlwood B.V., Francis M.J.O. (1972). The biosynthesis of monoterpenes. Chem. Rev..

[74] Schewe H., Mirata M.A., Holtmann D., Schrader J. (2011). Biooxidation of monoterpenes with bacterial monooxygenases. Process Biochem..

[75] Yassaa N., Peeken I., Zöllner E., Bluhm K., Arnold S., Spracklen D., Williams J. (2008). Evidence for marine production of monoterpenes. Environ. Chem..

[76] Giri A., Dhingra V., Giri C.C., Singh A., Ward O.P., Narasu M.L. (2001). Biotransformations using plant cells, organ cultures and enzyme systems: Current trends and future prospects. Biotechnol. Adv..

[77] Asghari G., Saidfar G., Mahmudi S. (2004). Biotransformation of aromatic aldehydes by cell cultures of *Peganum harmala* L. and *Silybum marianum* (L.) Gaertn. Iran. J. Pharm. Res..

[78] Simeo Y., Sinisterra J.V. (2009). Biotransformation of terpenoids: A green alternative for producing molecules with pharmacological activity. Mini. Rev. Org. Chem..

[79] Rasoul-Amini S., Fotooh-Abadi E., Ghasemi Y. (2011). Biotransformation of monoterpenes by immobilized microalgae. J. Appl. Phycol..

[80] Shimoda K., Kubota N., Hamada H., Kaji M., Hirata T. (2004). Asymmetric reduction of enones with *Synechococcus* sp. PCC 7942. Tetrahedron Asymmetry.

[81] Hamada H., Kondo Y., Ishihara K., Nakajima N., Hamada H., Kurihara R., Hirata T. (2003). Stereoselective biotransformation of limonene and limonene oxide by cyanobacterium, *Synechococcus* sp. PCC 7942. J. Biosci. Bioeng..

[82] Utsukihara T., Chai W., Kato N., Nakamura K., Horiuchi C.A. (2004). Reduction of (+)- and (−)-camphorquinones by cyanobacteria. J. Mol. Catal. B Enzym..

[83] Balcerzak L., Lipok J., Strub D., Lochyński S. (2014). Biotransformations of monoterpenes by photoautotrophic micro-organisms. J. Appl. Microbiol..

[84] Izaguirre G., Hwang C.J., Krasner S.W., McGuire M.J. (1982). Geosmin and 2-methylisoborneol from cyanobacteria in three water supply systems. Appl. Envir. Microbiol..

[85] Komatsu M., Tsuda M., Omura S., Oikawa H., Ikeda H. (2008). Identification and functional analysis of genes controlling biosynthesis of 2-methylisoborneol. Proc. Natl. Acad. Sci. USA.

[86] Giglio S., Chou W.K.W., Ikeda H., Cane D.E., Monis P.T. (2011). Biosynthesis of 2-methylisoborneol in cyanobacteria. Environ. Sci. Technol..

[87] Wang Z., Xu Y., Shao J., Wang J., Li R. (2011). Genes associated with 2-methylisoborneol biosynthesis in cyanobacteria: Isolation, characterization, and expression in response to light. PLoS One.

[88] Tung S.-C., Lin T.-F., Yang F.-C., Liu C.-L. (2008). Seasonal change and correlation with environmental parameters for 2-MIB in Feng-Shen Reservoir, Taiwan. Environ. Monit. Assess..

[89] Li Z., Hobson P., An W., Burch M.D., House J., Yang M. (2012). Earthy odor compounds production and loss in three cyanobacterial cultures. Water Res..

[90] Zimba P.V., Dionigi C.P., Millie D.F. (1999). Evaluating the relationship between photopigment synthesis and 2-methylisoborneol accumulation in cyanobacteria. J. Phycol..

[91] Kakimoto M., Ishikawa T., Miyagi A., Saito K., Miyazaki M., Asaeda T., Yamaguchi M., Uchimiya H., Kawai-Yamada M. (2014). Culture temperature affects gene expression and metabolic pathways in the 2-methylisoborneol-producing cyanobacterium *Pseudanabaena galeata*. J. Plant Physiol..

[92] Duetz W.A., Bouwmeester H., van Beilen J.B., Witholt B. (2003). Biotransformation of limonene by bacteria, fungi, yeasts, and plants. Appl. Microbiol. Biotechnol..

[93] Kiyota H., Okuda Y., Ito M., Hirai M.Y., Ikeuchi M. (2014). Engineering of cyanobacteria for the photosynthetic production of limonene from CO_2_. J. Biotechnol..

[94] Halfmann C., Gu L., Zhou R. (2014). Engineering cyanobacteria for the production of a cyclic hydrocarbon fuel from CO_2_ and H_2_O. Green Chem..

[95] Davies F.K., Work V.H., Beliaev A.S., Posewitz M.C. (2014). Engineering limonene and bisabolene production in wild type and a glycogen-deficient mutant of *Synechococcus* sp. PCC 7002. Front. Bioeng. Biotechnol..

[96] Bentley F.K., García-Cerdán J.G., Chen H.-C., Melis A. (2013). Paradigm of monoterpene (β-phellandrene) hydrocarbons production via photosynthesis in cyanobacteria. BioEnergy Res..

[97] Formighieri C., Melis A. (2014). Regulation of β-phellandrene synthase gene expression, recombinant protein accumulation, and monoterpene hydrocarbons production in *Synechocystis* transformants. Planta.

[98] Aprotosoaie A.C., Hăncianu M., Costache I.-I., Miron A. (2014). Linalool: A review on a key odorant molecule with valuable biological properties. Flavour Fragr. J..

[99] Cseke L., Dudareva N., Pichersky E. (1998). Structure and evolution of linalool synthase. Mol. Biol. Evol..

[100] Zhou R., Gibbons W. (2012). Genetically Engineered Cyanobacteria. U.S. Patent.

[101] Reinsvold R.E., Jinkerson R.E., Radakovits R., Posewitz M.C., Basu C. (2011). The production of the sesquiterpene β-caryophyllene in a transgenic strain of the cyanobacterium *Synechocystis*. J. Plant Physiol..

[102] Halfmann C., Gu L., Gibbons W., Zhou R. (2014). Genetically engineering cyanobacteria to convert CO_2_, water, and light into the long-chain hydrocarbon farnesene. Appl. Microbiol. Biotechnol..

[103] Chizzola R., Ramawat K.G., Mérillon J.-M. (2013). Regular monoterpenes and sesquiterpenes (Essential oils). Natural Products.

[104] Giglio S., Jiang J., Saint C.P., Cane D., Monis P.T. (2008). Isolation and characterization of the genes associated with geosmin production in cyanobacteria. Environ. Sci. Technol..

[105] Höckelmann C., Becher P.G., von Reuß S.H., Jüttner F. (2009). Sesquiterpenes of the geosmin-producing cyanobacterium *Calothrix* PCC 7507 and their toxicity to invertebrates. Z. Naturforsch. C..

[106] Orav A., Stulova I., Kailas T., Müürisepp M. (2004). Effect of storage on the essential oil composition of *Piper nigrum* L. fruits of different ripening states. J. Agric. Food Chem..

[107] Ghelardini C., Galeotti N., di Cesare Mannelli L., Mazzanti G., Bartolini A. (2001). Local anaesthetic activity of β-caryophyllene. Il Farmaco.

[108] Hendriks H., Malingre T.M., Batterman S., Bos R. (1975). Mono- and sesqui-terpene hydrocarbons of the essential oil of *Cannabis sativa*. Phytochem. Rep..

[109] Gertsch J., Leonti M., Raduner S., Racz I., Chen J.-Z., Xie X.-Q., Altmann K.-H., Karsak M., Zimmer A. (2008). Beta-caryophyllene is a dietary cannabinoid. Proc. Natl. Acad. Sci. USA.

[110] Prinsep M.R., Thomson R.A., West M.L., Wylie B.L. (1996). Tolypodiol, an antiinflammatory diterpenoid from the cyanobacterium *Tolypothrix nodosa*. J. Nat. Prod..

[111] Jaki B., Orjala J., Sticher O. (1999). A novel extracellular diterpenoid with antibacterial activity from the cyanobacterium *Nostoc commune*. J. Nat. Prod..

[112] Jaki B., Heilmann J., Sticher O. (2000). New antibacterial metabolites from the cyanobacterium *Nostoc commune* (EAWAG 122b). J. Nat. Prod..

[113] Pérez Gutiérrez R.M., Martínez Flores A., Vargas Solís R., Carmona Jimenez J. (2008). Two new antibacterial norabietane diterpenoids from cyanobacteria, *Microcoleous lacustris*. J. Nat. Med..

[114] Shpilyov A.V., Zinchenko V.V., Shestakov S.V., Grimm B., Lokstein H. (2005). Inactivation of the geranylgeranyl reductase (ChlP) gene in the cyanobacterium *Synechocystis* sp. PCC 6803. Biochim. Biophys. Acta.

[115] Huang Z.-R., Lin Y.-K., Fang J.-Y. (2009). Biological and pharmacological activities of squalene and related compounds: Potential uses in cosmetic dermatology. Molecules.

[116] Fox C.B. (2009). Squalene emulsions for parenteral vaccine and drug delivery. Molecules.

[117] Günes F.E. (2013). Medical use of squalene as a natural antioxidant. MÜSBED.

[118] Lee S., Poulter C.D. (2008). Cloning, solubilization, and characterization of squalene synthase from *Thermosynechococcus elongatus* BP-1. J. Bacteriol..

[119] Englund E., Pattanaik B., Ubhayasekera S.J.K., Stensjö K., Bergquist J., Lindberg P. (2014). Production of squalene in *Synechocystis* sp. PCC 6803. PLoS One.

[120] Siedenburg G., Jendrossek D. (2011). Squalene-hopene cyclases. Appl. Environ. Microbiol..

[121] Spanova M., Daum G. (2011). Squalene—Biochemistry, molecular biology, process biotechnology, and applications. Eur. J. Lipid Sci. Technol..

[122] Belin G.K. (2009). Investigation of hopanoid biomarkers in lake sediments by GC-MS and RP-HPLC-APCI-MS. E-J. Chem..

[123] Kannenberg E.L., Poralla K. (1999). Hopanoid biosynthesis and function in bacteria. Naturwissenschaften.

[124] Malott R.J., Steen-Kinnaird B.R., Lee T.D., Speert D.P. (2012). Identification of hopanoid biosynthesis genes involved in polymyxin resistance in *Burkholderia multivorans*. Antimicrob. Agents Chemother..

[125] Berry A.M., Harriott O.T., Moreau R.A., Osman S.F., Benson D.R., Jones A.D. (1993). Hopanoid lipids compose the *Frankia* vesicle envelope, presumptive barrier of oxygen diffusion to nitrogenase. Proc. Natl. Acad. Sci. USA.

[126] Welander P.V., Hunter R.C., Zhang L., Sessions A.L., Summons R.E., Newman D.K. (2009). Hopanoids play a role in membrane integrity and pH homeostasis in *Rhodopseudomonas palustris* TIE-1. J. Bacteriol..

[127] Schmerk C.L., Bernards M.A., Valvano M.A. (2011). Hopanoid production is required for low-pH tolerance, antimicrobial resistance, and motility in *Burkholderia cenocepacia*. J. Bacteriol..

[128] Hermans M.A., Neuss B., Sahm H. (1991). Content and composition of hopanoids in *Zymomonas mobilis* under various growth conditions. J. Bacteriol..

[129] Horbach S., Neuss B., Sahm H. (1991). Effect of azasqualene on hopanoid biosynthesis and ethanol tolerance of *Zymomonas mobilis*. FEMS Microbiol. Lett..

[130] Welander P.V., Doughty D.M., Wu C.-H., Mehay S., Summons R.E., Newman D.K. (2012). Identification and characterization of *Rhodopseudomonas palustris* TIE-1 hopanoid biosynthesis mutants. Geobiology.

[131] Schmerk C.L., Welander P.V., Hamad M.A., Bain K.L., Bernards M.A., Summons R.E., Valvano M.A. (2014). Elucidation of the *Burkholderia cenocepacia* hopanoid biosynthesis pathway uncovers functions for conserved proteins in hopanoid-producing bacteria. Environ. Microbiol..

[132] Jürgens U.J., Simonin P., Rohmer M. (1992). Localization and distribution of hopanoids in membrane systems of the cyanobacterium *Synechocystis* PCC 6714. FEMS Microbiol. Lett..

[133] Talbot H.M., Summons R.E., Jahnke L.L., Cockell C.S., Rohmer M., Farrimond P. (2008). Cyanobacterial bacteriohopanepolyol signatures from cultures and natural environmental settings. Org. Geochem..

[134] Doughty D.M., Hunter R.C., Summons R.E., Newman D.K. (2009). 2-Methylhopanoids are maximally produced in akinetes of *Nostoc punctiforme*: Geobiological implications. Geobiology.

[135] Doughty D.M., Dieterle M., Sessions A.L., Fischer W.W., Newman D.K. (2014). Probing the subcellular localization of hopanoid lipids in bacteria using NanoSIMS. PLoS One.

[136] Umeno D., Tobias A.V., Arnold F.H. (2002). Evolution of the C_30_ carotenoid synthase CrtM for function in a C_40_ pathway. J. Bacteriol..

[137] Domonkos I., Kis M., Gombos Z., Ughy B. (2013). Carotenoids, versatile components of oxygenic photosynthesis. Prog. Lipid Res..

[138] Havaux M. (1998). Carotenoids as membrane stabilizers in chloroplasts. Trends Plant Sci..

[139] Sozer O., Komenda J., Ughy B., Domonkos I., Laczkó-Dobos H., Malec P., Gombos Z., Kis M. (2010). Involvement of carotenoids in the synthesis and assembly of protein subunits of photosynthetic reaction centers of *Synechocystis* sp. PCC 6803. Plant Cell Physiol..

[140] Das A., Yoon S.-H., Lee S.-H., Kim J.-Y., Oh D.-K., Kim S.-W. (2007). An update on microbial carotenoid production: Application of recent metabolic engineering tools. Appl. Microbiol. Biotechnol..

[141] Takaichi S. (2011). Carotenoids in algae: Distributions, biosyntheses and functions. Mar. Drugs.

[142] Wang C., Kim J.-H., Kim S.-W. (2014). Synthetic biology and metabolic engineering for marine carotenoids: New opportunities and future prospects. Mar. Drugs.

[143] Estévez J.M., Cantero A., Reindl A., Reichler S., León P. (2001). 1-Deoxy-d-xylulose-5-phosphate synthase, a limiting enzyme for plastidic isoprenoid biosynthesis in plants. J. Biol. Chem..

[144] Kudoh K., Kawano Y., Hotta S., Sekine M., Watanabe T., Ihara M. (2014). Prerequisite for highly efficient isoprenoid production by cyanobacteria discovered through the over-expression of 1-deoxy-d-xylulose 5-phosphate synthase and carbon allocation analysis. J. Biosci. Bioeng..

